# Overview of Preventive Interventions Addressing Underage Drinking: State of the Evidence and Steps Toward Public Health Impact

**Published:** 2009

**Authors:** Richard Spoth, Mark Greenberg, Robert Turrisi

**Keywords:** Underage drinking, child, adolescent, preventive intervention, evaluation, efficacy assessment, effectiveness assessment, public health prevention model, literature review

## Abstract

Because underage drinking is a serious public health concern and associated with numerous detrimental consequences, many interventions to prevent underage drinking have been developed. However, the effectiveness of all these interventions has not been proven. A recent review of the relevant literature that used stringent criteria for the types of studies and interventions included, as well as for the evaluation and classification of the studies, found that out of more than 400 studies screened, only 127 could be evaluated for efficacy and only 41 showed some evidence of effects. In addition, several areas were identified in which intervention research could be strengthened. For example, increased coverage is needed for understudied areas of intervention (e.g., specific types of interventions or interventions in specific populations). Other aspects of the knowledge base in this area that can benefit from further improvement include, among others, the availability of longitudinal studies, availability of information on alcohol-specific outcomes, or availability of replication studies. The standards for determining and reporting evidence of effectiveness in different studies also need to be clarified. Finally, prevention research needs to adopt public health impact–oriented models to accurately determine the potential of existing interventions to prevent underage drinking and its consequences.

Underage drinking is a serious public health concern, as demonstrated by epidemiological data and results from studies investigating the social, health, and economic consequences of drinking by children and adolescents. According to the Monitoring the Future Survey (Johnston et al. 2006), 41.0 percent of 8th graders, 63.2 percent of 10th graders, and 75.1 percent of 12th graders in the United States reported that they already had consumed alcohol at some time in their lives, and 17.1 percent, 33.2 percent, and 47.0 percent, respectively, said they had consumed alcohol in the month preceding the survey. Even more troubling was the finding that 6.0 percent of 8th graders, 17.6 percent of 10th graders, and 30.2 percent of 12th graders surveyed reported that they had been drunk in the 30 days prior to the survey (Johnston et al. 2006), indicating that particularly harmful drinking patterns already are highly prevalent during adolescence.

As is the case with adults, alcohol consumption in adolescents is associated with a range of detrimental consequences:

- Adolescents who indulge in heavy drinking are more likely to engage in risky behaviors, such as drinking and driving. In fact, traffic crashes pose the single greatest mortality risk associated with underage drinking (Grunbaum et al. 2002; Hingson and Kenkel 2004; Hingson et al. 2005).
- Underage drinking contributes to both unintentional and intentional injury deaths among adolescents (Hingson and Kenkel 2004).
- Adolescents who drink heavily are at increased risk of short- and long-term physical health problems, such as sexually transmitted diseases resulting from unprotected sexual activity (Dee 2001; Grunbaum et al. 2002; Hingson et al. 2005; National Institute on Alcohol Abuse and Alcoholism [NIAAA] 1993).
- Adolescents who drink are at increased risk for behavioral problems, including delinquency, violence, and poor academic performance (Hingson et al. 2002; Spoth et al. 2006; Substance Abuse and Mental Health Services Administration [SAMHSA] 2008; Swartzwelder et al. 1995).
- Underage drinking is associated with mental health problems, such as depression and suicidality (NIAAA 1997; Windle 1992; Windle and Windle 2001).

Consequences of underage drinking result in substantial economic costs, as well as other less tangible costs to the adolescent, his or her family, the community, and society as a whole. Although no definitive data are available, the economic costs of underage drinking have been estimated to be more than $62 billion (Foster et al. 2003; Levy et al. 1999). Other important consequences of underage drinking include potentially lasting effects on brain structure and function that may interfere with the adolescent’s subsequent development (Tapert and Schweinsburg 2006; Tapert et al. 2008) and an increased risk of substance use during later adolescence as well as alcohol-related disorders (i.e., alcohol abuse and alcohol dependence)—and their associated problems—during adulthood (Grant and Dawson 1997).

All of these potential harmful consequences and associated costs underscore the importance of efforts to prevent underage drinking, using evidence-based preventive interventions, along with appropriate policy measures. However, intervening is challenging because of the following:

- Interventions must be designed and tested across developmental stages and a wide range of population subgroups considering that the needs and risk factors of, for example, a 10-year-old Caucasian boy living in a small town in Oklahoma likely differ considerably from those of a 15-year-old African-American boy living in the Bronx, or from those of an 18-year-old Hispanic girl living in south Florida.
- Interventions must be designed to reduce risk factors and promote protective factors that delay initiation of alcohol use, which, in turn, reduces harmful adolescent drinking patterns and the likelihood of developing alcohol-related problems during adulthood (Grant and Dawson 1997).
- A variety of effective interventions and policies operating at different levels must be developed, including comprehensive, community-level interventions.
- Interventions with demonstrated effectiveness must be effectively implemented on a large scale.

This article provides a comprehensive review of studies assessing the effectiveness of existing underage drinking interventions in adolescents in three developmental periods (i.e., less than 10 years of age, 10 to 15 years of age, and 16 to more than 20 years of age, excluding studies on college samples that have been reviewed elsewhere [Larimer and Cronce 2007]^1^). The goals of this review are to (1) highlight the compelling reasons for evidence-based preventive interventions targeting underage drinking, (2) review interventions that have shown evidence of effectiveness and efficacy,^2^ and (3) discuss key findings and their implications from a public health perspective.

## Methods of Intervention Selection and Evaluation

The existing literature on alcohol and other drug–related preventive interventions for underage drinking is vast. This article summarizes the results of a comprehensive critical review of this literature to assess the evidence for effectiveness of existing interventions (Spoth et al. 2008).

### Selection Criteria for Inclusion of the Interventions in the Analysis

Interventions included in the analysis reviewed here met the following criteria:

- *Scope of the intervention.* The analysis focused on universal interventions that target every member of an eligible population, selective interventions that are aimed at specific population subgroups which as a whole are at higher risk for alcohol use and alcohol-related problems, and indicated interventions that target specific individuals who have risk factors or conditions which identify them as being at risk for alcohol use and related problems. It did not include interventions targeting youth who already had an alcohol-related disorder.
- *Target population age.* The review included interventions targeting the three age-groups indicated above (i.e., less than 10 years, 10 to 15 years, and 16 to more than 20 years). Moreover, for the latter age-group, the review only includes those interventions aimed at high school students and young adults who do not attend college after high school. Interventions aimed at college students were excluded because other comprehensive reviews of these interventions are available.
- *Outcomes of interest.* The outcome measures that were required for an intervention to be included in the analysis differed according to the age-groups analyzed. For interventions aimed at youth aged 10 and older, the analysis included only those that incorporated outcome measures of alcohol use or abuse to determine the effectiveness of the intervention. Interventions whose outcome measures only included measures of illegal drug use, smoking, or broad measures of any drug use without directly measuring alcohol use or abuse were excluded. For interventions directed toward policy, law, or environmental changes, however, the availability of alcohol-specific outcomes was not required if data were available on outcomes that could be considered a logical consequence of alcohol use or abuse behavior (e.g., alcohol-related traffic incidents). For interventions aimed at children under age 10, different outcome measure criteria were used because these children rarely use alcohol. Therefore, the review included interventions aimed at reducing a key risk factor that is predictive of later alcohol use (e.g., early aggressive behavior) (Clark et al. 2005).

### Types of Intervention Literature Included in the Analysis

To ensure that all relevant evidence on existing interventions was included, three types of literature were candidates for analysis: (1) studies of specific interventions, (2) reviews and meta-analyses of outcome studies, and (3) summary reports of the evidence on specific interventions that were produced by agencies conducting evidence-based intervention reviews. These sources were identified through searches of literature databases of professional journals, reviews of relevant books and book chapters, literature reviews and meta-analyses, and searches of relevant Internet sources (i.e., the Web pages of appropriate agencies, such as the National Institutes of Health, Centers for Disease Control and Prevention, Office of Juvenile Justice and Delinquency Prevention, and others). Finally, for each new document obtained, the reference list was reviewed to identify any intervention that might have been omitted up to that point. Most reviews were completed by June 2006.

Together these sources yielded more than 400 interventions that were screened further, 127 of which provided at least some evidence concerning the desired outcome. Among those, 41 interventions met certain evaluation criteria (described in the next section) and therefore were included in the final analysis. These included 18 interventions for adolescents less than 10 years of age, 13 interventions for adolescents ages 10 to 15 years, and 10 interventions for those aged 16 to more than 20 years.

### Evaluation Criteria for Classification of Studies

To determine how promising the evidence for the effectiveness of a given intervention was, six criteria were established according to which of the interventions were evaluated. These included the following:

- *Experimental design.* Was the study a trial in which participants were randomly assigned to the intervention or a control condition? Or did the study design include an adequate comparison group?
- *Sample specification.* Was there sufficient information provided on the participants and their behavioral and social characteristics?
- *Outcome assessment.* Did the study include outcome data at a minimum of three different time points (i.e., before the intervention, after the intervention, and at a followup conducted at least 6 months after the primary intervention implementation or post-intervention assessment)?
- *Effects observed.* Was there a statistically significant difference in alcohol or alcohol-related outcomes?
- *Additional quality-of-evidence criteria.* Was there evidence that certain quality-of-evidence criteria established by the National Registry of Evidence-Based Programs and Practices (e.g., evaluation of sample attrition or appropriate statistical analyses [see SAMSHA 2008]) were met?
- *Manualization.* Was there a written manual that specified the target population and intervention procedures?

Based on an overall judgment of how well these criteria were met, the reviewed interventions then were classified into one of three categories: (1) most promising evidence, (2) mixed or emerging evidence, and (3) insufficient evidence or no evidence of effects. To be classified as most promising, the interventions had to meet all six criteria listed (see Spoth et al. 2008, for a more detailed description of the criteria). Because many of these criteria cannot easily be measured, and some criteria may be more important than others, the classification is based on an overall judgment of how well all criteria were met. Interventions that did not meet all the required criteria could be classified as mixed or emerging evidence. This was the case, for example, if the intervention demonstrated positive effects in some studies but no effects in other studies, or if within one study there were positive effects on some alcohol-related measures but no effects on other measures. Similarly, interventions that yielded positive effects, but for which the studies had substantial methodological limitations, and studies that found positive effects only for certain subgroups of the sample were classified as mixed or emerging evidence. Finally, all studies in which the intervention was aimed at adolescents younger than 10 years of age and that found an impact only on aggression, but not on later alcohol use, were classified as emerging.

Using this approach, a total of 12 interventions could be classified as most promising and 29 interventions as having mixed or emerging evidence; the remaining 86 interventions were classified as having insufficient evidence or no evidence of effects (see table 1). A brief summary of the most promising interventions is provided in table 2 (for additional information on studies with at least some evidence of effect, their samples, settings, results, and key sources, see Spoth et al. 2008). The reasons why most interventions were classified as having insufficient or no evidence of effects were wide ranging. For example, many studies had follow-up periods of less than 6 months; others demonstrated effects that were not statistically significant, had a weak experimental design, or failed to use alcohol-specific outcome measures.

## Key Findings and Their Implications

One of the main results of these analyses was the finding that of more than 400 interventions identified only 127 could be assessed for evidence of effectiveness. Further, of those 127 only about one-third demonstrated some evidence of positive effects, and of those about 10 percent could be classified as most promising. These findings suggest several topics for further discussion and investigation. One of these is the extent to which the existing evidence covers the different age-groups of adolescents analyzed, different domains or settings in which interventions can be delivered (e.g., family, school, community, and media), and different subgroups of adolescents (e.g., late teens, young adults not attending college, or minority populations). A second issue concerns the state of the art in intervention research—for example, what research designs are being used and should be used. Finally, the ways in which research results are reported in the literature also need to be addressed.

### Coverage of Understudied Areas of Intervention

This review of the evidence supporting the efficacy of existing interventions indicates that although much progress has been made in preventing alcohol use in underage populations, there still are gaps in understanding which type(s) of intervention, administered in which setting(s) and aimed at which target population(s), would be most effective in preventing underage drinking.

Researchers already have evaluated the relative advantages of different types of preventive interventions (i.e., universal versus selective versus indicated interventions). For example, Offord and colleagues (1998) discussed the key advantages of universal, selective, and indicated interventions and the trade-offs among them.^3^ This assessment concluded that a universal intervention would likely be preferable over targeted or indicated interventions if the problem it addresses (e.g., underage drinking) is highly prevalent and associated with high costs, whereas the intervention itself is relatively inexpensive and has been shown to be effective. In general, Offord and colleagues (1998) suggested a tiered strategy involving implementation of effective universal interventions, subsequent selective interventions for those who do not benefit sufficiently from the universal intervention, and indicated or clinical interventions for those who do not benefit from the selective intervention and are at high risk of problems.

Intervention findings can be summarized in numerous ways—for example, according to developmental period addressed (i.e., less than 10 years, 10 to 15 years, and 16 to more than 20 years), domain or setting in which the intervention is delivered (i.e., family, school, workplace, community, and multicomponent), or target population. The present review identified areas where evidence-based intervention is relatively stronger or weaker by focusing on coverage of the different developmental phases within key domains. In addition, the extent to which special populations and culturally based population subgroups were covered by the existing evidence also was taken into consideration.

#### Family-Focused Interventions

Several family-focused interventions have been developed, particularly for young children (i.e., preschool and primary school age) and for youths ages 10 to 15. These interventions typically address a range of risk and protective factors originating in the family, such as child monitoring, parent–child bonding, effective discipline, and parental involvement in child activities. Many of these interventions have been aimed at families with preschool-aged children and have focused on improving parent–child relationships, decreasing aggressive behavior, and enhancing the children’s social and cognitive competence for the transition to school. However, with few exceptions, studies evaluating these interventions have shown evidence primarily concerning effects on aggressive behavior, which is a risk factor for later alcohol use. Only one preschool program (i.e., the Nurse–Family Partnership) has shown positive effects on alcohol use when the participants reached their teen years (Olds et al. 1998; see Spoth et al. 2008).

In contrast, fewer family-focused interventions have been implemented and assessed with elementary school-aged children, particularly children in later elementary school years. In addition, some interventions for this age-group have integrated family and school components. Overall, several of these interventions have shown effectiveness in delaying alcohol use initiation, reducing alcohol use in the teenage years (e.g., Linking the Interests of Families and Teachers [Eddy et al. 2000, 2003], Seattle Social Development Project [Hawkins et al. 1991, 1992], Raising Healthy Children [Brown et al. 2005; Catalano et al. 2003], and the Preventive Treatment Program [Tremblay et al. 1996]) (see Spoth et al. 2008).

For adolescents ages 10–15, several family-focused interventions have shown considerable promise (e.g., Strengthening Families Program: For Parents and Youth 10–14 [Spoth et al. 2001, 2002, 2004, 2005], Guiding Good Choices [Park et al. 2000; Spoth et al. 2001, 2004], and Family Matters [Bauman et al. 2000, 2001, 2002; Ennett et al. 2001]). Interventions for this age-group can either be home based or administered in small groups, and the analysis indicates that small-group interventions show relatively stronger evidence of effect (see Spoth et al. 2008).

For older adolescents who are not going to college, no family-focused interventions with evidence of effectiveness could be identified, although other studies focusing on college-bound adolescents have shown some effectiveness (Larimer and Cronce 2007). Thus, this is an area where additional research is greatly needed.

#### School-Based Interventions

The area of school-based interventions has progressed significantly in recent years, and many interventions have been developed that include a variety of components, including life skills, peer refusal skills, role-playing to practice new skills, strengthening positive peer relationships, provision of accurate norms for alcohol and other drug use, and support to improve the adolescent’s emotional regulation. Such interventions have been shown to significantly reduce aggression and disruptive behavior in younger children, as well as early initiation and progression of use in younger and older adolescents.

However, there still are some important limitations to these studies. For example, studies of interventions aimed at elementary school children have shown effects primarily on the risk factor of aggressive behavior, rather than subsequent alcohol use itself, at least in part because they only followed participants for relatively short periods of time. Only a few studies monitored the participants into and through middle school (when alcohol use frequently is initiated) and thus were able to demonstrate effects on alcohol use. Additionally, there were no interventions with children in later elementary school years (i.e., grades 3 through 5) that focused on early alcohol use and which met the criteria for efficacy and effectiveness. Finally, only one school-based intervention targeted to high-school students could be classified as most promising and one could be classified as mixed or emerging evidence, indicating that interventions aimed at this age-group, which often is affected by harmful drinking patterns such as binge drinking, is an area requiring substantially more research.

### Multidomain Interventions

Multidomain interventions focus on two or more different domains of the child’s or youth’s life (i.e., the individual, family, school, worksite, community/environmental, or policy domain). This comprehensive approach is intended to maximize effectiveness. Because such interventions simultaneously address several aspects of the adolescents’ lives, they most often have focused on children of middle-school age or younger, who are less mobile and independent than older adolescents. Analyses of studies assessing these interventions have indicated that they can be effective, and some multicomponent interventions could be classified as most promising for the two younger age-groups considered here (e.g., the Prevention Treatment Program, which is aimed at children ages 10 or younger, and the Midwestern Prevention Project/Project STAR and Project Northland, which are aimed at adolescents ages 10–15). However, there still is a need for further research to develop and assess multicomponent interventions aimed at youths ages 16 and up. For example, such interventions could combine two interventions with proven efficacy from the school and family domains. In addition, challenges to large-scale implementation of these programs need to be addressed, including the extensive capacity and resources required for sustained, quality implementation.

#### Policy, Law, and Environmentally Focused Interventions

These types of interventions typically focus on older adolescents; the present analysis did not find any effective policy interventions aimed at adolescents below age 16 or 17, nor did it identify policy interventions that could be shown to delay initiation of alcohol use or reduce early alcohol use in these younger adolescents. For older adolescents, several interventions with some evidence of effect exist, including interventions focusing on reducing sales to minors, increasing identification checks by vendors, and reducing community tolerance of the sale of alcohol to minors and of underage drinking. However, these only could be classified as having mixed or emerging evidence of effect because they did not measure alcohol use outcomes or they involved too few communities to allow generalization of the findings. Thus, the design of studies assessing these interventions needs to be improved. Moreover, more attention should be focused on media-based interventions, especially because other studies support the influence of the mass media on underage drinking.

Another type of policy-, law-, and environmentally focused intervention that only could be classified as mixed or emerging evidence was the passage of laws raising the minimum drinking age. Some well-conducted studies have demonstrated that these laws can reduce rates of underage drinking (O’Malley et al. 1991), single-vehicle nighttime car crashes (Hingson 1983), and fatalities (Decker et al. 1988). Other studies (Ruhm 1996; Vingilis and Smart 1981), however, found no changes in the rates of crashes and fatalities after the introduction of such laws, so the evidence remains inconsistent. Moreover, it is unclear if these laws actually reduced underage drinking or if the adolescents merely drank in different venues (i.e., at home rather than in a bar) or obtained alcohol through different channels (i.e., through parents or older friends rather than purchasing it themselves) after the laws were passed. As a result, these measures could not be classified as having most promising evidence of effect.

### Conclusions

Despite substantial progress, the findings presented here indicate that there still is very limited research on interventions that target emerging alcohol use among late elementary school–aged children, high-school students, and older adolescents not currently in college. And although a wide range of domains were included in the analyses of interventions for high-school and post–high-school students, only a few theory-driven interventions targeted at this population could be identified. Moreover, although approximately one half of young adults ages 18–21 years in the United States (who tend to consume alcohol at high rates) do not attend college, few noncollege-based interventions exist that target this age-group. Thus, efforts to reduce underage drinking in this population are sorely missing, and additional work in this area is greatly needed.

### Need for Additional Coverage of Cultural Adaptations and Special Populations

This review has demonstrated that there already are several interventions with promising or emerging evidence which have been designed to address the special needs of minority populations and other understudied populations (e.g., youth living in rural areas) or which otherwise address cultural adaptations (e.g., Keepin’ It REAL). Nevertheless, the ability of many interventions to engage and influence youth from different cultural and ethnic backgrounds still needs to be enhanced, and for some developmental periods or target populations additional culturally specific interventions still need to be designed. Some of these changes may involve relatively superficial modifications, such as changes in wording, pictures, or stories to make the intervention more relevant to certain cultural groups. Other modifications, however, may affect the deeper structure of the intervention (e.g., the skills, attitudes, or policies to be conveyed) to meet the needs of different cultural groups. Finally, researchers need to demonstrate that already-proven, evidence-based models actually are effective across cultural groups.

### Key Issues in Current Intervention Research

In recent years, researchers have paid increasing attention to improving research methodology (e.g., study design and data analysis) for evaluating the effectiveness of prevention interventions. For example, more studies are using designs in which participants randomly are assigned to interventions or control conditions (i.e., randomized studies), which enhances the credibility of the observed outcomes. Nevertheless, many studies still have significant limitations and gaps that are reviewed below and which need to be addressed in future prevention research.

#### Limited Longitudinal Studies

One important issue is the implementation of rigorous studies that collect data over extended periods of time (i.e., longitudinal studies) to track both the initiation and progression of alcohol use. As mentioned earlier, many interventions targeting elementary school–aged children could not be included in the present review because their follow-up periods were too short. And even if some studies collected follow-up data for more than 6 months, very few provided data that were extensive and regular enough to examine the longer-term effects of the interventions. Moreover, initiation and early growth of alcohol use follow different patterns over time than do development of heavy drinking or binge drinking. Accordingly, it is critical to study both of these processes across early to later adolescent periods.

#### Specificity in Logical Models

Another concern is related to the rapid changes in alcohol use patterns during adolescence, which contributes to inconsistencies in findings across different assessments. For example, in some longitudinal studies, results of an intervention were mixed when data from different time points were compared or when different outcomes at one time point were compared. Because of this inherent variability and to distinguish it from variability resulting from suboptimal intervention or study designs, it is important that researchers clearly specify the logic of their intervention models, the objectives they wish to achieve (e.g., delay of initiation or prevention of regular alcohol use or binge drinking), and the specific intervention components designed to achieve these objectives.

#### Specificity in Self-Reported Outcome Measures and Related Issues

The utility and relevance of many prevention trials also is limited by the lack of outcome measures that specifically assess alcohol use (or use of specific other drugs). Thus, many trials assess substance use in general, without differentiating between alcohol, marijuana, and other illegal drugs. However, some interventions may be effective for preventing the use of one type of drug but not of others. Therefore, to adequately judge the effectiveness of interventions in preventing underage alcohol use, it is important that studies report alcohol-related outcomes separately from outcomes for other drugs. Moreover, more attention needs to be paid to the validity of the adolescents’ self-reported alcohol use because, for example, the settings in which self-reports are obtained have been shown to affect reporting (Azevedo et al. 2003).

#### Limited Replication Study

Another area of needed improvements concerns the independent replication of existing intervention outcome studies. In addition to simply confirming the initial effects observed for a given intervention, it would be useful to study the effects of systematic variations of the original intervention procedures. For example, one replication study of a school-based intervention found that the effectiveness varied depending on whether a teacher or another person implemented the intervention (St. Pierre et al. 2005). At the same time, clear guidelines need to be developed for such replication studies that specify, for example, to what extent the content of an intervention can be modified and a study of it still be considered a replication study (Hawkins 2004; St. Pierre et al. 2005).

#### Limited Study of Active Ingredients or Core Components and Outcome Mediators

As mentioned earlier, several of the interventions classified as most promising or mixed or emerging are multicomponent interventions that address more than one domain (e.g., family, school, and community). For these interventions, it may be useful to analyze in more detail which of the components are responsible for the observed effects and what capacity and resources they require for effective implementation. For example, using a strategy called outcome mediator modeling, researchers can identify the key mechanisms underlying observed effects by examining which components of the intervention account for substantial proportions of the variance in the targeted alcohol-related outcomes (Komro et al. 2001). Such analyses are particularly helpful in determining whether individual components differ in their effectiveness and/or whether different components enhance each other’s effectiveness (i.e., have synergistic effects). Similar analyses also can help clarify whether all of the components are necessary to achieve the observed positive effects or whether individual components might be just as effective on their own.

#### Limited Economic Analyses

Of the intervention studies reviewed here, only a few included any kind of economic analysis, such as an analysis of the economic benefits specific to alcohol-related outcomes. The economic analyses that have been conducted to date clearly indicate that several preventive interventions can be cost effective (Aos et al. 2004). Such analyses could be particularly important because if evidence-based interventions can be shown to yield alcohol-related cost savings, the dissemination of such interventions and their implementation in additional communities might be greatly enhanced.

#### Limited Study of Factors That Moderate Effects

It is essential to understand the factors that may moderate (i.e., increase or decrease) the magnitude of the effect of an intervention. This may be especially important in the case of universal interventions that target all members of a certain population. For example, is the intervention equally effective for all members of the population, regardless of their level of risk for alcohol-related problems? Relatively few analyses of this type have been conducted to date, primarily in the area of school-based interventions. However, such analyses are necessary so that in those cases in which the effects of an intervention are not uniform for all participants, the intervention design can be modified appropriately (Brown and Liao 1999; Dawson-McClure et al. 2004; Kellam and Rebok 1992).

#### Small Samples for Community, Policy, and Environmental Interventions

Overall, only a few studies of community-based interventions were identified that could be included in this analysis (e.g., Communities Mobilizing for Change on Alcohol [Wagenaar et al. 1999] or Community Trials Intervention to Reduce High-Risk Drinking [Grube 1997; Holder et al. 2000]). Moreover, the validity and generalizability of the findings of these studies is limited by the fact that most of them were conducted in a single community or a small number of communities, and sample sizes were small (Grube 1997; Holder et al. 2000). Thus, future studies of such interventions should involve larger numbers of communities as well as different types of communities (e.g., with respect to population size or ethnic composition and socioeconomic background of the population). Only under these conditions will researchers be able to identify specific factors that promote or inhibit the success for adapting such interventions to communities other than the ones that were part of the original study sample.

### Strong Consistent Standards for Evidence and Research Reporting

The key issues in intervention research listed above that should be addressed in future research of the effectiveness of intervention studies primarily pertain to changes in the design of the studies that can help improve assessment of the validity and generalizability of the results. As described below, another area of intervention research that can benefit from improvements is the development of more universal standards for evaluating interventions, conducting replication trials, and reporting the results.

#### Need for Consistency in and Broader Application of Evaluation Criteria

The number of published criteria used to evaluate evidence-based interventions has increased greatly in recent years. Unfortunately, these criteria vary considerably across publications. For example, one study (Lohr 2004) noted that various reviews of evidence-based medicine interventions used 20 different scales and 11 different checklists to evaluate the degree of efficacy. Because the criteria for the inclusion of effective programs vary, the standards and findings of different evaluations overlap only moderately at best (Mihalic 2004). One way to address such inconsistencies is standardized scoring of the quality of the evidence. Such an undertaking is highly challenging, however, because some quality-of-evidence criteria should be weighted more heavily than others (e.g., study design versus other factors of lesser relevance to study validity, such as participant expectations). Study designs differ substantially (e.g., simple designs evaluating a population before and after an intervention versus designs involving random assignment and multiple control groups). Moreover, the same quality criteria would have to be applied to all types of interventions across all phases of intervention research and across outcomes at all levels, which would not allow for adequate consideration of study-specific characteristics and objectives. Finally, it may be difficult to score specific evaluation criteria readily and reliably in studies with complex designs.

The evaluation of prevention programs in the area of substance abuse and mental health has improved greatly, but some need for improvements remains. It would be helpful if researchers consistently used widely accepted and rigorous criteria to assess the efficacy, effectiveness, and dissemination of preventive interventions, such as those developed by the Society for Prevention Research (Flay et al. 2005; Society for Prevention Research 2004). Although no single method can be used to assess all interventions, these standards note the importance of such general study design components as randomized trials (where feasible); using multiple, unbiased reporters; examining extensive follow-up effects; or fully reporting all outcome data. According to these standards, an intervention could be considered efficacious (i.e., had a positive effect under the controlled conditions of a clinical study) if it produced consistent and statistically significant findings in high-quality studies^4^ and the findings have some practical public health significance. To be considered effective (i.e., have an effect under “real-life” conditions), an intervention also would have to meet additional criteria. For example, the intervention would have to provide means (e.g., manuals or training programs) that allow it to be implemented by third parties and be evaluated under real-world conditions using a design that measures the levels of implementation and engagement of the target audience, demonstrates the practical importance of the outcome effects, and specifies the populations to which the findings can be generalized (Flay et al. 2005).

Although meeting the complete set of standards is a goal toward which researchers should aspire, it is recognized that few intervention research programs meet these standards (for example, availability of replication studies to demonstrate the efficacy of an intervention); accordingly, none of the interventions evaluated in the present analysis fully met these criteria for effectiveness. One of the greatest needs in prevention research is to conduct independent replication studies of existing programs to fully evaluate their efficacy and effectiveness.

#### Need for Improved Standards Concerning Intervention Replications

If additional replication studies of existing or new interventions are to be conducted, it also is critical to develop standards for judging when a study is truly a replication or when a program has been changed substantially without appropriate modification of the evaluation such that the subsequent study no longer qualifies as a replication study. For example, developers of intervention programs frequently refine their programs based on the results of initial outcome studies, and this refined version may then be disseminated to prospective customers and/or tested in additional studies. Under these circumstances, it would be beneficial to have standards specifying to what extent the study findings on the original version of the intervention still apply to the refined version and whether studies of the refined version can be considered replication studies for the original version of the intervention.

#### Need for Improved Reporting Standards

Great variation also exists in the way that data obtained in clinical trials are reported, and often these reports fail to include vital information. To help correct these reporting problems, an international group of scientists developed the Consolidated Standards of Reporting Trials (CONSORT) statement (available at: www.consort-statement.org/consort-statement/overview), which provides a 22-item checklist for the transparent reporting of randomized, clinical trials. It covers specific aspects of the various sections that typically are found in scientific reports of clinical studies (i.e., the background, methods, results, and discussion sections) and also provides a standardized diagram to show the progress of all trial participants from the time they enter the trial and randomly are assigned to a group until the time they leave the trial.^5^ These reporting standards have now been adopted by more than 150 medical and psychological journals, facilitating the evaluation and interpretation of study results published in those journals. A similar model called Transparent Reporting of Evaluations with Nonrandomized Designs (available at: www.trend-statement.org/asp/statement.asp) has been developed for reporting results of nonrandomized trials. Together with the Society for Prevention Research standards of evidence, these two models can substantially improve the validity and interpretability of the results of preventive intervention studies.

### Adopting Public Health Impact–Oriented Models

To have an impact on public health, there are several steps that should be taken. To begin, there is a need for development and testing of a broader range of interventions—across developmental phases, domains, and populations—so that the needs of all populations, particularly those that are underserved, are appropriately addressed. Importantly, in addition to testing and demonstrating favorable results on outcome measures, interventions also must be suitable for larger-scale implementation. The study of effective large-scale implementation needs substantial attention because only a small fraction of currently implemented interventions are evidence based, are implemented with high quality, and are used on a sustained basis (Federal Collaboration in What Works 2005; Spoth 2009). For example, quality frequently drops with sustained implementation, even for evidence-based interventions that initially are implemented with high quality. To translate effective interventions into widespread practice, it is essential that future analyses determine more clearly the factors that influence dissemination and sustained quality implementation of such interventions.

Studies already have identified some key factors that promote effective dissemination of evidence-based interventions and thereby help achieve a broad public health impact. These factors include, for example (Dearing 2004; Elliot and Mihalic 2004):

- The readiness and capacity of organizations to implement interventions;
- The quality of training and technical assistance available to the people administering the intervention; and
- Support from administrators in the implementation system.

Similarly, information is available on factors that influence the quality of implementation and its sustainability as well as on barriers to dissemination of public health interventions.

One approach to achieving public health impact is to adapt existing models of prevention intervention research. For example, the Institute of Medicine (Mrazek and Haggerty 1994) proposes that the design and initial testing of an intervention should be guided by theories on development and underlying factors related to a disorder. Based on the results of the initial tests, the intervention then should be refined before it is subjected to rigorous studies assessing its efficacy under controlled conditions. Subsequent replication studies and effectiveness studies under real-world conditions should determine the extent to which the intervention produces positive results with different populations and in different settings. Only after this comprehensive process has been completed would the intervention be ready for widespread dissemination. Although this model already provides many benefits, newly emerging models building on it seek to further enhance the dissemination and public health impact of preventive interventions. These models emphasize, for example, enhanced participation of communities as well as more consumer-oriented approaches during all stages of the research (Greenberg et al. 2003; Rotheram-Borus and Duan 2003; Sandler et al. 2005; Spoth et al. 2005). These emerging models incorporate private enterprise procedures for product development and marketing that take into consideration relevant consumer, provider, and funder issues and may thereby help optimize effective, broad- based dissemination. Other approaches incorporate community–university partnership models because some data have indicated that community teams supported by community–university partnerships can effectively engage potential participants in evidence-based interventions that can be implemented with high quality on a sustained basis, resulting in a range of positive, community-level outcomes (Spoth et al. 2007*a*,*b*).

Finally, as with other behaviorally oriented approaches to controlling a chronic disease, the translational aspect of intervention-related research needs to be emphasized. For example, basic causes of underage drinking (e.g., the role of peer and family influences on adolescents’ drinking behavior) are translated into real-world applications. To achieve this, researchers from various disciplines need to work together to stimulate the translation of science into practices that can have a public health impact and/or accelerate the rate at which effects of the interventions are seen at the population level (Spoth 2009); the Society for Prevention Research has developed a framework for advancing this type of translational research (see http://www.preventionresearch.org/).

Although some gaps still remain in the development, evaluation, and dissemination of interventions to prevent underage drinking, the existing and emerging models offer great promise for ultimately reducing the substantial public health burden of underage drinking and its associated impacts. To realize the full benefits of these approaches, however, it also is critically important that the necessary resources are available as well as sufficient funding.

## Conclusions

Over the past few decades, much progress has been made in the development of interventions to prevent underage drinking, and several interventions that can reduce the rate of alcohol use in underage populations and/or promote protective factors are now available. This progress has resulted, at least in part, from substantial methodological improvements in study design and analysis, such as the increased evaluation of interventions using stringent randomized clinical trials. Despite this progress, however, the full potential of such preventive interventions has not yet been reached. For example, as described in the preceding sections, not all developmental stages, population subgroups, and intervention domains are adequately covered by existing interventions. Evidence and reporting standards also warrant further improvements, as do intervention research models and strategies to enhance dissemination and quality of implementation, and sustainability of evidence-based interventions in real-world settings. Finally, to achieve greater public health impact it will be essential to mobilize sufficient resources to provide the infrastructure and capacity necessary to support research on and high-quality, sustained implementation of interventions at all levels.

## Figures and Tables

**Table 1:** Interventions Aimed at Different Age-Groups of Adolescents With Some Level of Evidence of Effect

	**Age-Group**		

**Level of Evidence**	**<10 Years of Age**	**10–15 Years of Age**	**16 to >20 Years of Age**
**Most promising evidence**	Linking the Interests of Families and Teachers Raising Healthy Children Seattle Social Development Project Nurse–Family Partnership Program Preventive Treatment Program (Montreal)	Keepin’ It REAL Midwestern Prevention Project/Project STAR Project Northland Strengthening Families Program: For Parents and Youth 10–14	Project Toward No Drug Abuse Yale Work and Family Stress Program Mississippi Alcohol Safety Education Program and Added Brief Individual Intervention
**Mixed or emerging evidence**	Classroom-centered Intervention Families and Schools Together Fast Track First Steps to Success Good Behavior Game I Can Problem Solve Olweus Bullying Prevention Perry Preschool Program Promoting Alternative Thinking Strategies Schools and Families Educating Children Second Step The Incredible Years Triple-P-Positive Parenting	Bicultural Competence Skills Program Family Matters Families That Care: Guiding Good Choices (formerly Preparing for the Drug-Free Years) Healthy School and Drugs Life Skills Training New Beginnings Program Project Alert School Health and Alcohol Harm Reduction Project SODAS City	Athletes Training and Learning to Avoid Steroids Brief Motivational Intervention in Emergency Department Communities Mobilizing for Change on Alcohol Community Trials Intervention to Reduce High-Risk Drinking Problem Drinking in Workplace Raising minimum drinking age law (State-level) Raising minimum drinking age law

NOTE: For a description of the various interventions and their evidence, see Table 2 and Spoth et al. 2008.

**Table 2A:** Summary of Preventive Interventions Classified As Most Promising Targeting Adolescents in Three Different Age-Groups

**Children Less Than 10 Years of Age**					
**Intervention**	**Type**	**Domain**	**Sample/Ethnicity/Setting**	**Main Results**	**Sources**
Linking the Interests of Families and Teachers	Universal	Family, school	6 schools, 651 students grades 1 through 5 Primarily White College town	Grade 1 intervention: effects on child physical aggression Grade 5 intervention: effects on patterned alcohol use across grades 6 through 8	Eddy et al. 2000, 2003 www.oslc.org
Raising Healthy Children	Universal	Family, school	10 schools, 989 students grades 1 through 7 Primarily White Suburban	Reductions in teacher reports of disruptive and aggressive behavior in grade 2; no effects by parent reports in grade 2 Reduction in growth of alcohol use No reduction in alcohol initiation rates	Brown et al. 2005; Catalano et al. 2003 depts.washington.edu/sdrg
Seattle Social Development Project	Universal	Family, school	18 schools, 810 students grades 1 through 5 Multiethnic Urban	Grade 2: effects on school-age aggression (White boys only) Grade 5: effects on alcohol initiation At age 18: reductions in heavy drinking	Hawkins et al. 1991, 1992 depts.washington.edu/sdrg/page4.html#ssDP
Nurse–Family Partnership Program	Selective	Family	300 pregnant women White Rural	Mothers: reduced behavioral problems attributable to alcohol and other drug use Children: fewer days of alcohol consumption at age 15	Olds et al. 1998 www.nursefamilypartnership.org
Preventive Treatment Program (Montreal)	Selective	Multicomponent	166 children grades 1 through 2 with early behavioral problems French–Canadian Urban	At age 15: significant effects on drinking to the point of being drunk	Tremblay et al. 1996 www.gripinfo.ca/Grip/Public

NOTE: For more information on these results, see Spoth et al. 2008.

**Table 2B:** Summary of Preventive Interventions Classified As Most Promising Targeting Adolescents in Three Different Age-Groups

**Adolescents Ages 10–15**					
**Intervention**	**Type**	**Domain**	**Sample/Ethnicity/Setting**	**Main Results**	**Sources**
Keepin’ It REAL	Universal	School	35 public schools, 4,235 students Multiethnic Urban	At 19 months after program implementation: lower increases in past-month alcohol use Three conditions were tested that represented different versions of the same program	Hecht et al. 2003 www.dsgonline.com/mpg2.5/TitleV_MPG_Table_Ind_Rec.asp?=634id
Midwestern Prevention Project/Project STAR	Universal	Multicomponent	42 public middle and junior high schools, 3,412 students White and Black Urban	At 1-year followup: significant effects on proportion of students reporting past-week and past-month alcohol use Secondary effects on baseline use persisted up to 1.5 years after baseline, not beyond	Pentz et al. 1989, 1990; Pentz and Valente 1995; Chou et al. 1998
Project Northland	Universal	Multicomponent	24 school districts Multiethnic Tribal, urban, rural	Intervention in grades 6 through 8: significantly lower past-month and past-week use compared with control group at 2.5 years after baseline Intervention in grades 11 through 12: significantly less binge drinking compared with control group at 6.5 years after baseline	Perry et al. 1996, 2002; Klepp et al. 1995 http://ibs.colorado.edu/cspv/wwa/cgi-bin/progdetails.pl?progid=65
Strengthening Families Program: For Parents and Youths 10–14	Universal	Family	Study 1: 33 public schools, 667 students Primarily White Rural Study 2: 36 public schools, 1,650 students Primarily White Rural	Study 1: significantly lower rates of initiation in each of three alcohol lifetime or new-users measures at 4 years after baseline compared with control subjects Lifetime use, drunkenness, and time to initiation significantly lower at 6 years after baseline Study 2: when combined with Life Skills Training program, significantly less alcohol initiation at 1.5 years after baseline; slower growth in weekly drunkenness at 2.5 years after baseline	Spoth et al. 2001, 2002, 2004, 2005 www.strengtheningfamiliesprogram.org http://ibs.colorado.edu/cspv/wwa/cgi-bin/progdetails.pl?progrid=235

**Table 2C:** Summary of Preventive Interventions Classified as Most Promising Targeting Adolescents in Three Different Age-Groups

**High-School Students or Older Participants Ages 16 to More Than 20 Years**					
**Intervention**	**Type**	**Domain**	**Sample/Ethnicity/Setting**	**Main Results**	**Sources**
Project Toward No Drug Abuse	Selective and indicated	School	42 schools, 2,468 high-school students Multiethnic Southern California	Reduced levels of alcohol use among baseline users at 1-year followup Reduced number of drinks per month at 22-month followup	Sussman et al. 2002
Yale Work and Family Stress Project	Universal	Workplace	4 job sites, 239 secretarial employees Primarily White Connecticut-based corporations	Problem drinkers benefited more from enhanced program in reducing DUI recidivism	Snow et al. 2002
Mississippi Alcohol Safety Education Program and Added Brief Individual Intervention	Indicated	Community	4,074 adjudicated first-time DUI offenders (primarily male) 36 percent minorities Mississippi	Women tended to have lower rates of recidivism and higher rates of depressed mood than did men	Wells-Parker and Williams 2002

NOTE: For more information on these results, see Spoth et al. 2008

## Footnotes

1 These age-groups are those that are targeted by the NIAAA Underage Drinking Initiative.

2 The efficacy of an intervention relates to whether the intervention worked under the ideal and controlled conditions of a prevention trial, with patients who were selected based on certain criteria and who were monitored to ensure that they received the intervention as prescribed, and in which the intervention was given by well-trained providers who followed the prescribed protocol. In contrast, effectiveness relates to whether the intervention worked under relatively more “real-life” conditions, with participants from a variety of backgrounds who may or may not have followed all the instructions and where the intervention was given by providers who may or may not have followed the regular protocol for the intervention.

3 Trade-offs considered in the analysis by Offord and colleagues (1998) included the prevalence and costs of the problem to be addressed, effectiveness of the intervention, extent to which the intervention reached those who needed it, quality of the intervention (including compliance rates), and costs of the intervention.

4 In general, these findings should be demonstrated and consistent in at least two independent studies.

5 This kind of information is useful for judging the efficacy of an intervention because when a study demonstrates that an intervention is effective with most participants who complete it but that, for example, 80 percent of participants drop out of the trial before it is completed, that intervention needs to be judged differently than an intervention that may be effective in a smaller proportion of participants but in which almost all participants have completed the study.
